# Brief cognitive behavior therapy for stigmatization, depression, quality of life, social support and adherence to treatment among patients with HIV/AIDS: a randomized control trial

**DOI:** 10.1186/s12888-023-05013-2

**Published:** 2023-07-25

**Authors:** Qasir Abbas, Mehru Nisa, Muhammad Umar Khan, Nida Anwar, Sumayah Aljhani, Zoobia Ramzan, Mafia Shahzadi

**Affiliations:** 1 Department of Applied Psychology, Government College University Faisalabad, Main Campus, Faisalabad, Punjab Pakistan; 2 Dow International Medical College, Dow University of Health Sciences, Karachi, Pakistan; 3 Department of Psychiatry, College of Medicine, Qassim University, Buraydah, Saudi Arabia

**Keywords:** Cognitive behavior therapy, Stigmatization, Depression, Quality of life, Social support, Treatment adherence, Patients with HIV/AIDS

## Abstract

**Objective:**

Individuals living with HIV/AIDs are at a high risk of many problems like depression, stigma, quality of life, decreased adherence to treatment, and lack of social support. The present study aimed to investigate the impact of brief-cognitive behavior therapy (B-CBT) on reducing depression and stigma and improving treatment adherence, quality of life, and social support among patients with HIV/AIDS attending antiretroviral therapy (ART).

**Materials and methods:**

This randomized clinical trial was conducted at ART Clinic in the Tehsil Headquarters Hospital Shahkot Nankana Sahib from July 2021 to October 2021. After baseline screening, 126 patients met the eligibility criteria and 63 were allocated to the experimental group (EXPg = 63) and 63 to waitlist-control group (WLCg = 63). Participants’ age range was from 20 to 55 years. Participants who were taking ART treatment were enrolled for the CBT treatment. Before this, all the participants completed a baseline assessment to ensure a level of severity and diagnosis. A total of eight CBT based therapeutic sessions were conducted individually with EXPg. To assess the outcomes among patients receiving ART, we used Demographic form, Patient health questionnaire, HIV stigma scale, General medication adherence scale, Multidimensional scale of perceived social support, and WHOQOL BREF scale.

**Results:**

Findings suggest that B-CBT significantly reduced the level of depression (i.e. F (1, 78) = 101.38, *p* < .000, η2 = .599), and social stigma (i.e. F (1, 78) = 208.47, *p* < .000, η2 = .787) among patients with HIV/AIDS. Furthermore, CBT substantially improved the level of adherence to treatment (i.e. F(1,78) = 24.75, *p* < .000, η2 = .503), social support (i.e. F (1, 78) = 128.33, *p* < .000, η2 = .606), and quality of life (i.e. F (1, 78) = 373.39, *p* < .000, η2 = .837) among patients with HIV/AIDS. Significant mean difference M(SD) on PHQ at post-analysis in the EXPg vs. WLCg was seen 1.22(0.47) vs. 2.30(0.68) and similarly, on MPSS at a post-analysis in the EXPg vs. WLCg 2.85(0.36) vs. 1.70(0.51) which indicates sound therapeutic outcomes.

**Conclusions:**

Cognitive behavioral therapy effectively decreases the level of depression and stigma and enhances the level of social support, quality of life, and adherence to treatment among HIV/AIDS patients. It is concluded that cognitive behavior therapy is an effective treatment approach for patients with HIV/AIDS.

**Trial registration:**

Thai clinical trial registry (i.e. TCTR = TCTR20210702002).

**Supplementary Information:**

The online version contains supplementary material available at 10.1186/s12888-023-05013-2.

## Introduction

HIV/AIDS is a life-long chronic disease with many potentially debilitating symptoms that can reduce one’s quality of life [1, 2]. It leads to severe sickness or death [3]. Aside from the symptoms, patients with HIV/AIDS also face various cultural and psychosocial issues (like stigma); this combined suffering makes them increasingly prone to developing depression,,being more stressed and losing hope [4]. Stigma is common in HIV-positive people, diminishing their quality of life and leading to poor clinical outcomes [3]. Internalized HIV-related Stigma may lead to HIV transmission-related risky behaviors such as poor antiretroviral adherence, unsafe sex, avoidance of medical treatment, and a reluctance towards HIV status disclosure in situations where it could pose a risk to themselves or to others [5]. Defining HIV-related Stigma (HRS) is difficult because of the interplay of various factors like personal cognitions, structural inequalities, cultural differences, discrimination by health care providers, and a general fear of discrimination [6]. Internalized Stigma refers to endorsing negative beliefs and misconceptions about HIV/AIDS [7–11].

According to a report published by the United Nations’ programme on HIV/AIDS in 2020, HIV/AIDS remains a major public health concern around the world; As per the aforesaid report, 37.7 million people were living with HIV/AIDS,, 1.5 million people were newly infected with it, 680 000 people died from its related illnesses, and 27.5 million people were accessing antiretroviral therapy worldwide. According to the report people living with HIV/AIDS in Asia and the Pacific were around 5.8 million, with 240,000 new HIV/AIDS infections, 130 000 AIDS-related deaths, and 3.7 million accessing treatment. According to the same report, in Pakistan, the number of adults and children living with HIV/AIDS were 200,000 with HIV/AIDS prevalence rate of around 0.2% in the age bracket of 15 to 29 years. and adult and child deaths due to AIDS were 8200, whereas, people living with HIV/AIDS who know their status were 45,000with 24,000 currently receiving ART [12].

One of the major problems usually accompanying HIV/AIDS is major depression (MD); it is highly prevalent in HIV/AIDS infected patients and this psychiatric disorders could increase mortality this strata [13–16] Depression is characterized by a depressed mood, lack of concentration, diminished energy, disturbed sleep, and low self-esteem [17]. There is a an increased risk of HIV/AIDS transmission when the patient does not adhere to ART [18]. ART increases life expectancy and quality of life [19–21]. ART can also reduce the rate of transmission from the patients to others [22]. Depression is a key predictor of poor adherence to HIV/AIDS medication [23]. Social support can be described as the social, psychological, and interpersonal assistance that optimizes health and well-being of an individual’s [24]. Perceived social support has been linked with better QOL among people living with HIV [25, 26]. Among patients with HIV/AIDS, a positive association between increased social support and better clinical outcomes has been found [27–29].

Psychotherapy appears to be an effective treatment for adult depression [30, 31]. However, cognitive behavioral approaches to psychotherapy have been found to specifically improve antiretroviral medication adherence and reduce depression in patients with HIV/AIDS [32, 33]. Cognitive-Behavioral Therapy (CBT) has emerged as a highly promising intervention; it is a goal-oriented therapy that focuses on modifying negative thought patterns and behaviors, and by enhancing emotion regulation, problem-solving skills, and positive coping mechanisms [34]. Furthermore, CBT interventions are beneficial in increasing a person’s stress coping methods repertoire and, as a result, improving mental health in HIV patients [35, 36]. Adherence to HIV treatment, self-care, and medication can be improved through CBT therapies [30, 32, 37, 38]. Applying cognitive-behavioral techniques enhances medication adherence by addressing negative thinking, fostering adaptive coping skills, and promoting effective problem-solving; this approach helps patients manage their medication side effects and HIV-related stress, resulting in improved health outcomes [38]. Spies et al. [39] found that i cognitive-behavioral therapy (CBT) is effective in reducing depression and improving antiretroviral medication (ART) adherence in patients with HIV/AIDS [32, 39–42]. By combining behavioral and cognitive techniques, CBT targets activity and thinking patterns. [43, 44]. CBT is effective in enhancing social support for HIV/AIDS patients by strengthening support networks and improving interpersonal skills [45, 46]. Both individual and group psychotherapies are effective for reducing depressive symptoms in people with HIV [47]. However, there is a need for a briefer, time-bound, and solution-focused version of psychotherapy for psychological betterment of patients of serious illnesses that does not drain their valuable resources of time and money too much [48–50]. Such a modality of psychological intervention is highly needed in underdeveloped countries like Pakistan owing to limited financial resources available to the patients here [51, 52]. Brief CBT is one such modality of psychotherapy that addresses the psychosocial concerns in only a few number of sessions (usually around 10) and is time-bound [53]. We developed an 8 session version of CBT tailored for the needs of patients with HIV/AIDS by utilizing a combination of classic and brief CBT literature [53, 54].

This study was aimed to see how brief CBT affects HIV patients’ psychological health and adherence to ART, because, usually, when people get to know about their HIV positive status, they feel shame. And as HIV/AIDS diagnosis is not considered in a supportive manner in Pakistani society and the families of the diagnosed patient change attitude towards the patient for the worse, so that’s why they face many problems [55]. The patients with HIV/AIDS usually internalize the stigma attached to the disease and usually feel mentally disturbed which makes them vulnerable to the development of different psychiatric problems. Therefore, they need psychological treatment. Their quality of life is also affected due to potential HIV/AIDS progression, poor treatment adherence, a lack social support, and comorbid psychiatric problems. In the present study, psychological treatment was given to HIV/AIDS patients in the form of cognitive behaviour therapy to address different problems of theirs. It was aimed at enhancing mental health, decreasing their stigma and depression, and increasing their treatment adherence, social support, and quality of life.

## Materials and methods

### Study setting

This prospective clinical trial was conducted at Tehsil Headquarter (THQ) hospital providing diagnostic and treatment facilities to patients with HIV/AIDS in Shahkot, Nankana District (Punjab, Pakistan). This was a public sector hospital which has ART clinic that provides free treatment facilities to patients with HIV/AIDS.

### Research design

This study was a prospective, randomized control trial (RCT) conducted from 25 May 2021 to December 2022 at a hospital clinic where individuals who have HIV/AIDS are provided with treatment. In this study, we compared the effectiveness of cognitive behavior therapy between experimental and waitlist control groups using pre-and post-test design. The Institutional Review Board of Government College University, Faisalabad, Pakistan, approved the study protocol (IRB) (i.e. Ref.No. GCUF/ERC/2267, dated 17/06/2021). This clinical trial was registered in the Thai clinical trial registry (i.e. TCTR = TCTR20210702002, with URL: https://www.thaiclinicaltrials.org/show/TCTR20210702002) with first registration date 02/07/2021.

### Participants

**Fig. 1:**
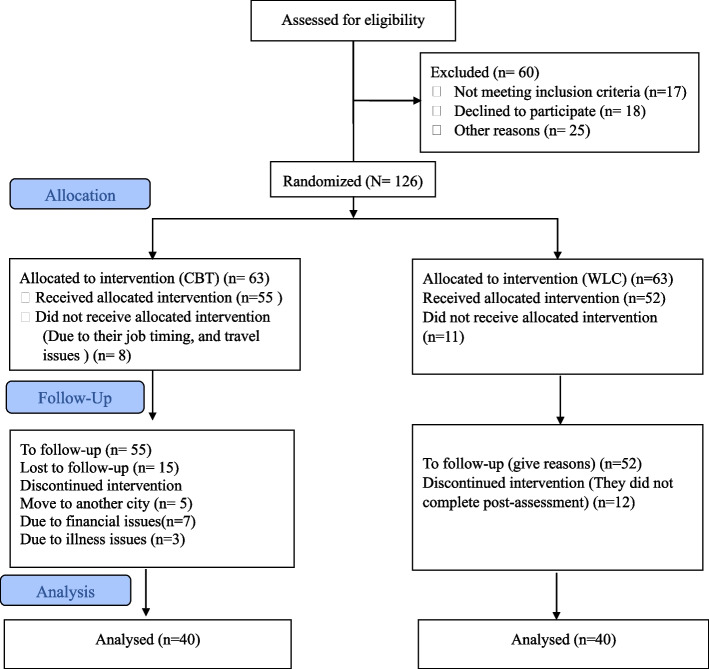
Flowchart diagram of patients with HIV/AIDs

A total of 186 participants diagnosed with HIV/AIDs were recruited for eligibility from THQ Hospital Shahkot, Pakistan. We calculated sample size using G-Power software estimating effect size (f) = 0.20, α = 0.05, power (1-β error prob.) = 0.95 with actual power = 0.96 which structured sample size of 48 (for both experimental and waitlist control conditions) [56]. Initially, participants were screened using Patient Health Questionnaire-9, and those who scored ≥ 5 (at least with mild symptoms severity) were enrolled in the treatment. Sixty participants were excluded;; a) 18 patients were excluded due to their comorbid condition with other physical ailments) 12 patients were excluded due to a lack of history of treatment and non-provision of information c) 13 were excluded because their traveling time between their clinic and their home was long and they could not visit the clinic as often as needed, and d) 17 patients were excluded who showed a lack of interest in the study (*n* = 17). Therefore, 126 out of 186 met the inclusion criteria and were then allocated in a ratio of 1:1 to our experimental (*n* = 63) and waitlist-control (*n *= 63) groups (see Fig. 1).

### Inclusion and exclusion criteria

In this RCT, diagnosed patients having HIV/AIDS were recruited. The first eligibility criterion was that the enrolled participants must be registered with the ART clinic and must also be receiving ART regularly. Patients’ HIV/AIDs diagnosis was made on the basis of medical reports and evaluation. Participants who had initiated their treatment using ART and had completed all HIV/AIDS related medical evaluations, were recruited for this study. Participants were then assessed by our clinical psychologist. The further eligibility criteria were as follows: a) participants availing out-door patients’ facility with mean score of ≥ 5 on the PHQ-9 (at least having mild severity of depression or higher) and b) the participants were then cross-checked against set exclusion criteria, thereby excluding pregnant women, participants with substance use disorder, individuals with any physiological comorbidity, and individuals who were availing in-patient services. Participants with any other serious comorbidity (i.e., any other serious physiological or psychiatric disease except depression) were excluded from the study. Furthermore, individuals under the age of 20 and over the age of 55 years were also excluded from the study.

### Assessment measures

#### Demographic form

A detailed demographic form and history taking form were used to get personal information (i.e., age, education, socioeconomic level, family system, family members, marital status, employment status, risk transmission, and total earning members), history of illness (i.e. duration of HIV/AIDS, duration of taking current medication, age at the time HIV/AIDS diagnosis, previous treatments, a family member died due to HIV/AIDS, any other family member diagnosed with HIV/AIDS, information about HIV/AIDS).

#### Patient Health Questionnaire (PHQ-9)

The PHQ-9 was originally developed by Spitzer et al. It was translated and validated into an Urdu version by Ahmad et al., (Cronbach’s alpha = 0.91) [57, 58]. The Urdu version of the PHQ-9 was used in this study. The PHQ is a nine-item self-report measure based on the DSM diagnostic criteria of depressive disorder. It investigates about the presence of different symptom sets of major depressive episodes that were present in an individual during the past two weeks. The PHQ-9 uses a 4-point response scale ranging from not at all (0) to nearly every day (3). Higher scores on the scale indicate the severity of depressive symptoms, and lower scores indicate low severity. The PHQ-9 also provides symptom severity, i.e., none-minimal (0–4), mild (5–9), and moderate (10–14), moderate-severe (15–19) and severe (20–27) respectively. Furthermore, many studies have shown its legitimacy and reliability [57, 58].

#### HIV Stigma Scale (HSS)

HSS is a 40-item scale was originally developed by Berger et al. [59]. It was translated and validated into an Urdu version by Saif and Shehzad [60]. The Urdu version of HSS was used in the present study. The HSS assesses four factors i.e., personalized stigma, disclosure concerns, negative self-image, and public attitudes. Each item is scored using a 4-point Likert-type scale ranging from “strongly disagree” to “strongly agree”. The HSS scores range from 40 to 160. The higher scores indicate the high intensity of stigma. The HSS has good psychometric properties and is a valid and reliable measure [60].

#### General Medication Adherence Scale (GMAS)

GMAS is originally developed in Urdu Language was used [61]. The GMAS consists of 11 items with four possible options divided into three domains. The first domain is non-adherence due to patient behavior (NAPB), the second domain is non-adherence due to other diseases and pill burdens (ADPB), and the third domain is nonadherence due to financial constraints (NAFC). Each item's score ranges from 0 to 3, and the total sum of all items yields an overall adherence score, which is categorized as high (30–33), good (27–29), partial (17–26), low (11–16), and poor adherence (≤ 10). The GMAS has good reliability of 0.80 and test–retest reliability of 0.996, respectively [61].

#### Multidimensional Perceived Social Support Scale (MPSS)

MPPS was originally developed by Zimet et al. [62]*.* It was translated and validated into an Urdu version by Akhtar et al. [63].The Urdu version of MSPSS was used in the current study. The MPSS is a 12-item, unidimensional questionnaire that assesses how people view their social support system which includes their family, friends, and significant others. Each item is scored on a 7-point scale ranging from “strongly disagree” to “strongly agree”. A higher score indicates a higher level of social support. The overall reliability of MPSS is > 0.80 and for subscale 0.82, 0.86, and 0.86 respectively.

#### WHO Quality of Life Scale-Brief (WHQOL-B)

Urdu version of WHQOL-B was used [64]. The Urdu version of WHQOL-B was used in the present study. WHQOL-B contains two items to assess overall QOL and 24 items for general health having four major domains i.e., physical health, psychological health, social relationships, and environmental health. Each item is rated on a 5-point Likert scale ranging from 1 to 5. Higher scores indicate a higher quality of life. The Urdu version of WHQOL-B has an internal consistency of 0.86 and for subdomains is 0.78, 0.75, and 0.73, respectively.

### Randomization

All the respondents were randomly allocated to experimental and waitlist-control groups with an equal ratio of 1:1. To achieve this goal, we generated a randomization schedule using permuted block randomization so that each block contained a random assignment to each of the two groups (see Fig. 1).

### Interventions

#### Antiretroviral Therapy (ART)

Antiretroviral therapy (ART) reduces HIV replication and the infection of new cells and improves immune system function. ART has dramatically reduced HIV-related morbidity and mortality; as a result, the life expectancy of individuals with HIV/AIDS is increasing [65]. HIV is a debilitating disease; however, antiretroviral treatment (ART) helps promote effective viral suppression, reduces transmission risk, and prevents death [66]. In the present study, we did not actively provide ART therapy per se, however, we only took those patients with HIV/AIDS who were already receiving ART therapy as our participants.

#### Brief-cognitive behavior therapy (B-CBT)

We developed an 8-session brief CBT or B-CBT to be an effective treatment addressing psychiatric symptoms and psychoeducation. Patients with HIV/AIDS usually perceive stigma, decreased quality of life and emotional disturbance, leading to low motivation and a lack of interest in treatment. Our B-CBT aimed at effectively dealing with depressive symptoms, enhancing motivation, and reducing stigma through psychoeducation, cognitive restructuring, skill training, stress management, and relapse prevention [31]. Our B-CBT was aimed to be helpful for patients with HIV/AIDS in order to educate them about the significance of medication and to motivate them to cope with their emotional problems, ultimately enhancing treatment adherence [30]. Other studies have also suggested that B-CBT has proved to be a feasible, acceptable, and promising treatment approach for patients with HIV/AIDS [41]. Our B-CBT also aimed at being an effective psychotherapy to address emotional disturbance among HIV/AIDS patients within 8 session timeframe [33]. The treatment protocol of our 8-session B-CBT was designed as a synthesis of the classic CBT and other B-CBT approaches [53, 54] (See Supplementary File-Table A).

### Procedure

In this study, all procedures were approved by the Institutional Review Board (IRB) of Government College University, Faisalabad. We assigned the participants to each treatment condition through randomization to ensure the numbers allocated to each group were comparable and balanced. Participants were recruited from the OPD of THQ Hospital, Shahkot. One of our psychotherapists would start-off by briefly explained to the subjects about the study. Participants would then be tentatively included if thy gave verbal consent. Then, the therapist would describe confidentiality, and a consent form would be given to the participants to sign. After signing the informed consent form, the participants would proceed towards the initial assessment process. Then (after initial assessment and eligibility check) the patients with HIV/AIDS would be randomly assigned to treatment conditions (i.e., B-CBT) and waitlist control. Then we would use different scales to determine the treatment outcomes at pre- and post-assessment i.e., PHQ-9, HIVSS, GMAS, MSPSS, WHOQOL BREF.

### Statistical analysis

Descriptive statistics (Mean &SD) were used to calculate sample demographic characteristics. We used t-test and chi-square test of the scales used at baseline assessment stage to make the group matchable and comparable on the variables of concern. After the conclusion of the trial, repeated measures ANOVA was used to investigate the differences between groups, time, and group x time interaction. Frequency distribution statistics were also used to find out the depression and level of social support after treatment. An alpha of 0.05 was used for all analyses, and *p*-values < 0.05 were submitted to Bonferroni correction using IBM SPSS Statistics (Version 26).

## Results

### Recruitment and attrition

In this clinical trial, 186 respondents were assessed for eligibility; 60 out of 186 were excluded due to inclusion and exclusion criteria, and 126 met the inclusion criteria then they were allocated to the EXPg (*n* = 63(50%) and WLCg (*n* = 63(50%)) through random assignment (see Fig. 1). We investigated no significant difference at baseline between the EXPg vs. WLCg. Participants were all literate people (the word matric. is a short form of the word matriculation which means 10^th^ grade in Pakistan). Participants’ age range was from 20 to 55 years including both men (*n* = 52) and women (*n* = 74) with single (*n* = 19) and married (*n* = 107) marital status. Baseline discrepancies were all insignificant among demographic characteristics and were as follows: age, monthly income, age at diagnosis, gender, education, birth order, marital status, family system, reason of HIV/AIDs, duration of HIV/AIDs diagnosis, duration of HIV/AIDs medication, family members died due to HIV/AIDs, any other family member diagnosed with HIV/AIDs, information about HIV/AIDs, and medication dose irregularity (see Table 1).

**Table 1:** Comparison of participants’ demographic characteristics group-wise and overall

Variables	Category	Overall	Groups			
Characteristics			Experimental	Control	χ2/t	p
N Allocated		126	63(50.0%)	63(50.0%)		
N Dropout		46	23(50.0%)	23(50.0%)		
N Final		80	40(50.0%)	40(50.0%)		
Age		126	31.87(8.35)	31.93(8.53)	-.042	.846
Monthly Income M(SD)		126	16,857.14(8153.48)	15,476.19(6951.11)	1.023	.380
Gender	Male (n%)	52	25(39.7%)	27(42.9%)	.131	.717
	Female (n%)	74	38(60.3%)	36(57.1%)		
Education	< Matric.(n%)	72	35(55.6%)	37(58.7%)	.376	.829
	Matric. (n%)	33	18(28.6%)	15(23.8%)		
	> Matric. (n%)	21	10(15.9%)	11(17.5%)		
Birth Order	First (n%)	26	14(22.2%)	12(19.0%)	1.004	.605
	Other (n%)	80	41(65.1%)	39(61.9%)		
	Last (n%)	20	8(12.7%)	12(19.0%)		
Marital Status	Married (n%)	107	52(82.5%)	55(87.3%)	.558	.455
	Single (n%)	19	11(17.5%)	8(12.7%)		
Family System	Nuclear (n%)	77	41(65.1%)	36(57.1%)	.835	.361
	Joint (n%)	49	22(34.9%)	27(42.9%)		
Reason of HIV/AIDS	Don’t Know (n%)	81	38(60.3%)	43(68.3%)	1.052	.591
	Used syringe (n%)	37	20(31.7%)	17(27.0%)		
	Blood Transfusion (n%)	8	5(7.9%)	3(4.8%)		
Duration of HIV/AIDS diagnosis	1 Month to 1.5 years (n%)	83	41(65.1%)	42(66.7%)	.155	.925
	< 2.5 year (n%)	36	18(28.6%)	18(28.6%)		
	> 3.5 years (n%)	7	4(6.3%)	3(4.8%)		
Duration of HIV/AIDS medication	1 Month to 1.5 years (n%)	84	42(66.7%)	42(66.7%)	.171	.918
	< 2.5 year (n%)	35	17(27.0%)	18(28.6%)		
	> 3.5 years (n%)	7	4(6.3%)	3(4.8%)		
Age at diagnosis		126	30.59(8.11)	30.67(8.47)	-.054	.766
A family member died due to HIV/AIDS	Yes (n%)	117	57(90.5%)	60(95.2%)	1.077	.299
	No (n%)	9	6(9.5%)	3(4.8%)		
Any other family member diagnosed with HIV/AIDS	Yes (n%)	79	41(65.1%)	38(60.3%)	.305	.581
	No (n%)	47	22(34.9%)	25(39.7%)		
Information about HIV/AIDS	Don’t Know (n%)	106	51(81.0%)	55(87.3%)	.951	.329
	Know (n%)	20	12(19.0%)	8(12.7%)		
Medication dose Irregularity	No (n%)	81	39(61.9%)	42(66.7%)	.311	.577
	Yes (n%)	45	24(38.1%)	21(33.3%)		

Findings show that there is a significant mean difference in the baseline scores and post-testing scores among two groups on PHQ-9 which reflects that almost 0.60% CBT reduced the symptoms severity of depressive symptoms in the EXPg. Similarly, significant mean scores difference was found between EXPg and WLCg on PSS, DSS, NSI, PAS, and overall HIV stigma scale. This indicates that CBT played substantial role to address PSS 0.66%, DSS 0.73%, NSI 0.77%, PAS 0.68%) and HIVS 0.79%. In addition, significant difference was seen on GMAS which reflects that 0.51% CBT enhanced the level of medical adherence in EXPg as compared to WLCg. Findings supported that CBT positively enhanced the degree SOS 0.46%, FAS 0.45%, FRS 0.32% and overall social support 0.61% in EXPg. Furthermore, treatment condition produced significant difference on GHS 0.46%, PHS 0.66%), PSS 0.59%, SRS 0.77%, ESN.66% and overall QOLB 0.83% respectively (see Table 2).

**Table 2:** Mean (standard deviation) and Repeated Measure Design of clinical scores during pre- and post-test intervention

*Groups*					*Repeated Measure ANOVA*						
*Experimental Group*			*Waitlist Control*		*Group*		*Time*		*Group x Time*		***η***_***p***_^***2***^
	*Baseline* *M(SD)*	*Post- Test* *M(SD)*	*Baseline* *M(SD)*	*Post- Test* *M(SD)*	*F*	*p-* *Value*	*F*	*p-Value*	*F*	*P-* *Value*	
PHQ-9	11.70(4.23)	3.10(2.79)	9.92(3.42)	9.62(3.20)	13.19	.001	116.57	.001	101.38	.001	.599
PSS	57.92(5.65)	42.67(4.41)	56.52(5.37)	54.35(2.54)	45.87	.001	148.61	.001	83.67	.001	.656
DSS	32.27(2.48)	21.72(2.67)	32.87(2.57)	32.10(1.93)	214.26	.001	206.08	.001	153.53	.001	.725
NSI	42.07(5.31)	22.87(3.93)	40.50(4.01)	39.90(2.92)	124.66	.001	261.14	.001	230.44	.001	.770
PAS	65.05(6.33)	48.12(5.05)	63.70(4.86)	61.25(2.73)	54.88	.001	162.19	.001	90.52	.001	.675
HIVS	128.50(9.12)	87.32(8.28)	126.85(9.22)	123.52(6.27)	171.90	.001	288.11	.001	208.42	.001	.787
NAPA	13.65(2.19)	16.43(2.82)	13.70(2.19)	13.65(1.99)	9.18	.003	27.66	.001	29.73	.001	.262
NADP	11.73(2.45)	14.43(2.28)	11.75(2.46)	11.83(2.49)	5.88	.018	173.66	.001	155.39	.001	.690
GMAS	25.38(3.06)	30.85(3.78)	25.45(2.96)	25.47(3.02)	16.56	.001	78.79	.001	77.36	.001	.503
SOS	4.31(1.57)	6.58(.47)	4.30(1.44)	4.85(.89)	19.12	.001	66.71	.001	24.87	.001	.461
FAS	2.76(1.20)	5.71(1.20)	3.00(1.25)	2.55(1.12)	45.19	.001	63.50	.001	117.44	.001	.449
FRS	2.66(1.38)	5.10(1.68)	2.44(1.16)	2.21(.91)	45.23	.001	36.36	.001	53.25	.001	.318
MSSS	3.25(.82)	5.80(.74)	3.25(.95)	3.20(.67)	87.30	.001	119.83	.001	128.33	.001	.606
GHS	5.72(1.28)	9.00(.93)	6.00(1.19)	4.95(.87)	88.26	.001	66.65	.001	251.83	.001	.461
PHS	14.00(1.47)	17.85(1.28)	14.36(1.77)	13.18(1.08)	76.92	.001	150.22	.001	71.11	.001	.658
PSS	13.30(1.86)	17.62(1.70)	13.96(1.98)	12.30(1.28)	79.14	.001	110.28	.001	21.82	.001	.586
SRS	12.05(1.66)	18.35(1.74)	11.65(1.74)	10.30(1.39)	222.11	.001	265.77	.001	111.27	.001	.773
ESN	14.85(1.36)	17.38(1.09)	15.31(1.55)	13.66(1.14)	51.99	.001	128.40	.001	5.73	p001	.662
QOLB	71.65(6.02)	93.05(3.51)	73.80(7.83)	65.75(4.13)	156.97	.001	373.39	.001	76.73	.001	.827

*η*_*p*_^*2*^ Partial Squared Eta, *PHQ* Patient Health questionnaire, *PSS* Personalized Stigma Subscale, *DSS* Disclosure Subscale, *NSI* Negative Self Image, *PAS* Public Attitudes Subscale, *HIVS* HIV Stigma Scale, *NAPA* Non-adherence due to Patient Behavior, *NADP* Non-adherence due to Additional Disease and Pill Burden, *GMAS* General Medication Adherence Scale, *SOS* Significant other subscales, *FAS* Family Subscale, *FRS* Friends Subscale, *MSSS* Multidimensional Social Support Scale, *GHS* General Health Subscale, *PHS* Physical Health Subscale, *PSS* Psychological Subscale, *SRS* Social Relationship Subscale, *ENS* Environmental Subscale, *QOLB* Quality of Life Brief

Findings indicate a significant mean difference (i.e., M(SD) on PHQ at post-analysis in the experimental group vs. waitlist control (i.e., 1.22(0.47) vs. 2.30(0.68). Moreover, participants who got CBT eventually had a significant decrease in the severity of their symptoms on PHQ. For example, at the pre-test, the number of participants that had mild symptoms was 3(7.5%), moderate 13(32.5%), moderate-severe 17(42.5%), and severe 7(17.5%), whereas, at post-test the number of participants who had mild symptoms was 32(80.0%), moderate 7(17.5%), moderate-severe 1(2.5%) and nobody was there with severe symptom severity. This indicates a significant change (reduction) in symptoms severity when comparing pre- and post-test scenarios. In contrast, the participants' symptoms severity remains almost the same in the waitlist control when seen in relation with pre- and post-test. Similarly, a significant mean difference was observed in MPSS at post-test in the experimental group vs. waitlist control (i.e., 2.85(0.36) vs. 1.70(0.51), which indicates that participants had an increase in their level of perceived social support after treatment. For example, in the pre-test, participants perceived low 17(42.5%) and moderate 23(57.5%) social support, while in the post-test, 6(15%) participants perceived moderate social support and 34(85%) perceived high social support but in the waitlist-control group remain almost same score at pre- and post-test scenarios (see Table 3).

**Table 3:** Comparison of depression and social support levels of severity group-wise and overall

*Groups*				
*Characteristics*	*Experimental*		*Waitlist Control*	
	*Pre-Test*	*Post-Test*	*Pre-Test*	*Post-Test*
	*M(SD)*		*M(SD)*	
PHQ M(SD)	2.70(.85)	1.22(.47)	2.42(.67)	2.30(.68)
Mild (%)	3(7.5%)	32(80.0%)	1(2.5%)	4(10.0%)
Moderate (%)	13(32.5%)	7(17.5%)	24(60.0%)	21(52.5%)
Mod. Severe (%)	17(42.5%)	1(2.5%)	12(30.0%)	14(35.0%)
Severe (%)	7(17.5%)	–	3(7.5%)	1(2.5%)
MPSS M(SD)	1.57(.50)	2.85(.36)	1.57(.50)	1.70(.51)
Low support (%)	17(42.5%)	–	17(42.5%)	13(32.5%)
Mod. Support (%)	23(57.5%)	6(15.0%)	23(57.5%)	26(65.0%)
High Support (%)	–	34(85.0%)	–	1(2.5%)

*PHQ* Patient Health Questionnaire, *MSS* Multidimensional Perceived Social Support Scale

**Fig. 2:**
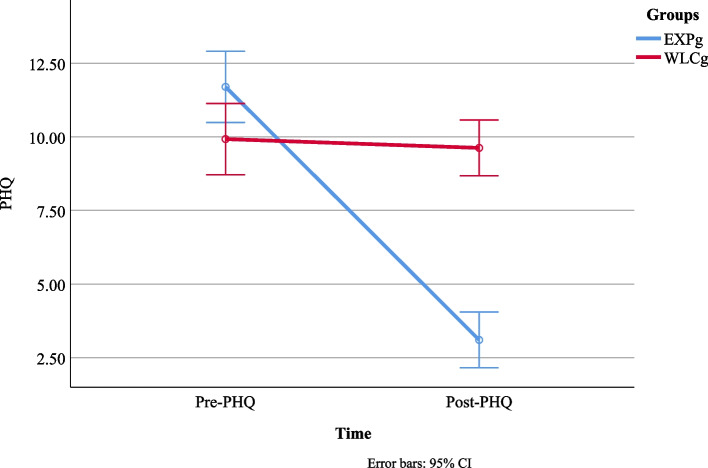
Score difference of pre and post-test analysis on PHQ between EXPg vs. WLCg

Results show that depression was significantly reduced on PHQ after receiving B-CBT intervention in HIV/AIDS patients, and post-test analysis showed a significant change in depressive symptoms. Patients reported improvement in the pre-and post-testing of PHQ-9 among the experimental group. On the other hand, no significant difference was found on the pre and post-testing of PHQ-9 among the control group. It indicates that intervention was effective for reducing depression among HIV/AIDS Patients (Fig. 2).

**Fig. 3:**
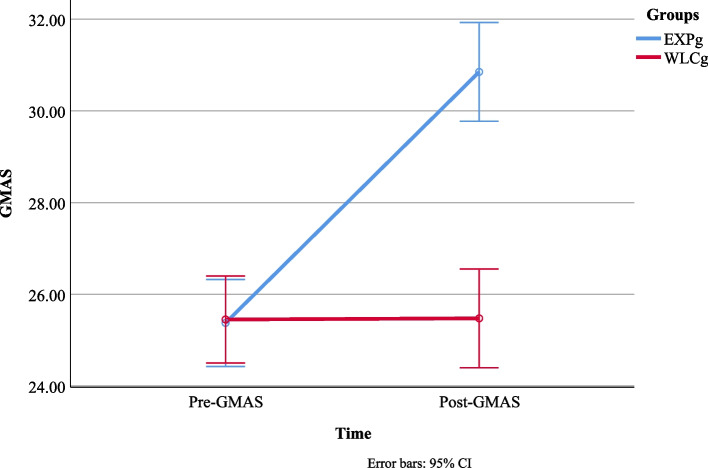
Score difference of pre and post-test analysis on GMAS between EXPg vs. WLCg

Results show that medication adherence was significantly increased on GMAS after receiving CBT intervention among HIV/AIDS patients, and post-test analysis shows a significant change in medication adherence. Patients reported improvement in the pre- and post-testing of GMAS among the experimental group. On the other hand, no significant difference was found on the pre- and post-testing of GMAS among the control group. It indicates that intervention was effective for enhancing medication adherence among HIV/AIDS Patients (Fig. 3).

## Discussion

In this clinical trial, we noted that depressive symptoms can be reduced through B-CBT, and concurrently, treatment adherence to ART can also be improved. B-CBT substantially helped address various mental health problems of patients with HIV/AIDs. This indicates that a combination of medicinal treatment (ART) with a psychological intervention (B-CBT) is a better treatment choice for treating patients with HIV/AIDS as compared with care as usual [41]. Patients with depressive symptoms were seen as more reluctant toward treatment due to a lack of interest and hopelessness in life; they were less motivated to take part in treatment, but we observed that B-CBT significantly reduced their distress, and they showed interest in treatment and started to live with positive hopes towards life [67, 68]. Alongside HIV/AIDs and related medical problems, mental health problems also play a significant role in treatment and recovery. For example, lack of social support, social stigma, family attitude, marital issues, financial strains, and treatment advancement can cause depression and ultimately treatment non-adherence [69–71]. B-CBT protocol helped the patients by providing psychoeducation, creating awareness, developing hope toward life, and reducing emotional disturbance through the therapeutic process using different techniques and skill training.

Moreover, our study’s findings indicate that reduction in depressive symptoms and perceived stigma significantly improved treatment adherence [72, 73]. Stigma is seen as a primary concern for people living with (HIV/AIDS), and this has a great negative impact on their quality of life and family relationships, which is a significant cause of psychiatric problems [74]. People with HIV/AIDs feel suppressed and guilty because of their illness, which hurts them internally and this could lead them towards depression and stigmatization [75]. CBT remained an effective treatment to improve treatment adherence [76, 77]. We observed that some patients were not using proper medications and missed or delayed the recommended doses due to a lack of awareness and misconceptions about the medication and treatment outcomes; after B-CBT, significant positive change in patients’ attitudes and behavior toward treatment was observed. Some other issues, such as the number of medications, complex dosage schedules, and medication side effects reduce treatment adherence. After the B-CBT sessions, significant change in these problems was observed [78, 79].

Furthermore, study findings reported that participants perceived low social support at the baseline due to social stigma and depressive symptoms. However, after B-CBT treatment, participants perceived a significant positive change in social support [80, 81]. When we educate the patients and train them through role plays, they try to look forward to different opportunities of social connection, they try to improve their social support system, and they also try to talk to others to become more comfortable with them. This could be the reason of reduction in their stigma and depression [82, 83]. Increasing social support among patients also tends to improves quality of life and communication with friends and family members; thereby decreasing the number of depressive symptoms and perceived self-based stigma [84, 85]. During B-CBT treatment, we planned different activities to engage the patients in social communication to reduce their perceived stigma and loneliness; these things brought a positive change in participants’ attitudes and behavior. We also designed some other helpful activities for them like: priority setting, decision making, life skill training, and muscle-relaxation training etcetera [80, 86].

Furthermore, when the patients felt relaxed mentally, and believed that medication and therapeutic training are important to manage daily routines and treatment procedures, they took their medication regularly and increased their interest in the treatment [42, 87]. The aspects we observed among the participants who received B-CBT were significantly different compared to WLC [88]. B-CBT appeared to be a successful treatment intervention in increasing quality of life and satisfaction with life and reducing anxiety and depression symptoms [89, 90]. Findings also reveal that B-CBT was an effective therapy to improve the quality of life and overall psychological well-being of patients with HIV/AIDs [91].

## Limitations of the study

This study has valuable insights; however, it also has some limitations. In this study, only those patients who were availing outpatient facility were included, and patients in the inpatient setting were excluded from the study. Participants with medical comorbidities were also excluded; therefore, the findings of this study might not be applicable to the patients with HIV/AIDS with other medical comorbidities. Furthermore, in Pakistan, psychological treatment is not very common and is not frequently used in many settings; therefore, most of the Pakistani population is not familiar with this kind of treatment. This was a major limitation when we tried to talk with the patients. Most of them did not know what psychological treatment is, and some left the treatment just because there was no medication involved in it for their psychological complaints. Another limitation of this study was that pregnant women were not studied.

### Recommendations

Overall, the study discovered the impact of cognitive behavior therapy on reducing stigma and sadness and improving social support, quality of life, and treatment adherence among patients with HIV/AIDS on antiretroviral therapy (ART). According to our study, it is hereby recommended to note which other factors influence the development and strengthening of therapeutic alliances during HIV/AIDS treatment. Also, it should also be explored why do patients terminate their treatment?. Further, the complex interaction between transference and therapeutic alliance is not clear. We tried to explore this complex relationship during treatment. However, it was beyond the reach of any therapist to find all factors that may influence an outcome at a time, so further research should be done to investigate the other factors.

Further research may include more patients in the form of a larger sample size. Further, most of the previous studies were done in Western countries, and very few people in Pakistan know how to manage the disease, so there still is a need to conduct studies in the south Asian culture, especially in underdeveloped countries like Pakistan. Hence, further research is required in these places because the rate of depression and psychological distress, negative thinking, add to the suffering of the in patients with HIV/AIDS, and also because the disease is spreading at a higher pace than before. The overall current study showed good and fruitful results. It is important to note patients, with some help from a psychotherapist, can independently manage their problems and the level of depression through cognitive behavioral therapy. Further research work on the ART side-effects that result in medication and a need to manage it is needed.

### The implication of the study

This study could be used in hospitals to help doctors treat patients with HIV/AIDS and to promote the knowledge and management skills to address mental health problems. Therefore, to improve patients' mental health and wellness, we must create a separate place/counseling room in each HIV/AIDS hospital where patients' confidence can be maintained. This study is conducted with HIV/AIDS patients, and it is the first clinical trial in Pakistan; the study findings should be important for policymaking, preparing treatment protocols, and treating patients with HIV/AIDS. The study provides the basis for introducing psychological or counseling intervention to address the mental and physiological health of patients with HIV/AIDS.

## Conclusion

This clinical trial successfully addressed many psychological problems and low adherence to treatment using B-CBT in patients with HIV/AIDS who were availing ART treatment in a hospital. B-CBT significantly reduced depressive symptoms and emotional disturbance, which maybe were, due to social stigma, hence, a substantial improvement was seen in social support and quality of life. Furthermore, B-CBT produced significant and reliable outcomes addressing mental health problems using effective modalities to guide the patients and understand the significance of psychological treatment addressing the problems of patients with HIV/AIDs and has shown to be a supportive intervention of choice for such patients.

## Supplementary Information

**Additional file 1.** 

## Data Availability

The dataset generated and/or analyzed during the present study is not publicly available because no permission was taken from the participants and the hospital administration where the study was conducted. The datasets are available from the corresponding authors upon request.
