# Supplementary material for: Brief cognitive behavior therapy for stigmatization, depression, quality of life, social support and adherence to treatment among patients with HIV/AIDS: a randomized control trial
Source: BMC Psychiatry. 2023 Jul 25;23:539. doi: 10.1186/s12888-023-05013-2 (PMC10367308; doi:10.1186/s12888-023-05013-2)
Supplement: Supplementary file 1 — Additional file 1. [file 12888_2023_5013_MOESM1_ESM.docx]

**Intervention and Goals:**

Structured intervention program for patients with mental health problems, who availed cognitive behavior therapy and who availed both medication and cognitive behavior therapy is given.

**Table A**

| ***Intervention--Cognitive Behavior Therapy Protocol*** | | |
| --- | --- | --- |
| ***Sr. No*** | ***Agenda*** | ***Goals Intervention Strategies and outcome*** |
| 1 | ***Psychoeducation*** | ***Goals***  Feedback about assessment  Educate about HIV  Educate about HIV, How it effects on your psychological Health  To educate the patient about the depression symptoms  To educate the patient how stigma increase depression and decrease quality of life  Educate about HIV  Educate about HIV, How it effects on your psychological Health Provide rationale for CBT based treatment(Paulus et al., 2020).  Increased patient motivation for treatment(Chattopadhyay et al., 2017). |
| 2 | ***Behavioral Activation*** | ***Goals***  Interviewing the patient about daily routine(Chattopadhyay et al., 2017).  Helping them to become more active and to give themselves credit for their efforts  Bringing about realistic, structured behavioral changes in small steps (Chattopadhyay et al., 2017).  Exploring the relationship between activity and mood.  Encourage engagement in activities (Berg et al., 2008).  To help patients select activities and made a specific plan.  Fostered use of social support(Berg et al., 2008).  Increased awareness and involvement into activities(Berg et al., 2008). |
| 3 | ***Cognitive Conceptualization and Cognitive Restructuring*** | ***Goals***  To identify and recognize the cognitive distortions  To explore the automatic thoughts and emotions about illness  Evaluate and respond to thoughts, emotions, and beliefs  Automatic thought records were used by patients with some help from therapist (Chattopadhyay et al., 2017).  The cognitive errors were corrected using individual cognitive strategies(Chattopadhyay et al., 2017). |
| 4 | ***Treatment Adherence Training*** | ***Goals***  Give exact details on the regimen, as well as drug interactions and adverse effects (Dima et al., 2013).  provide Motivation involves both personal and social components, as well as one's views and attitudes regarding the consequence of adherence (Dima et al., 2013).  Behavioral skills for individuals to adhere to one’s regimen and one’s self-efficacy in using those skills (Dima et al., 2013).  Psychoeducation about medicine resistance(Chattopadhyay et al., 2017).  Plan for coping with side effects of medications and medical regimen(Safren et al., 1999).  Formulate a daily schedule for medication and other self-care behaviors(Safren et al., 1999).  Develop cues for taking medications or implementing other self-care procedures(Safren et al., 1999).  Talk about the risk of not taking medication(Safren et al., 1999). |
| 5 | ***Stigma Reduction and Stress Management*** | ***Goals***  To improve ability to effectively cope with stress (Brown & Vanable, 2008).  To improve stress management skills (Brown & Vanable,2008).  Emphasized skill development to cope with HIV-related stressors (Zhang et al., 2021).  To change their conception of HIV (Tshabalala & Visser, 2011).  To change their sense of self-worth and to empower them with more adaptive ways of thinking(Tshabalala & Visser, 2011).  To deal with their experience of stigma(Tshabalala & Visser, 2011).  Given the stigma associated with HIV and the social groups HIV disproportionately affects, it is essential to use CBT/MI skills to address stigma and its impact on well-being and HIV-related self care (Harkness et al., 2020). |
| 6 | ***Problem solving*** | ***Goals***  Define problem(Chattopadhyay et al., 2017).  Evaluation of alternatives(Chattopadhyay et al., 2017).  Decision making about solution(Chattopadhyay et al., 2017).  Implementation of solution(Chattopadhyay et al., 2017).  Increased ability to break problems down(Berg et al., 2008).  Solve barrier that interfere with medication adherence(Brandt et al., 2018).  Make specific plans to overcome these barriers(Brandt et al., 2018).  Collaborate with the client to identify how their syndemic problems (i.e., depression, family rejection, unemployment) are impacting their overall well-being and HIV-related self-care (Harkness et al., 2020). |
| 7 | ***Risky Behavior Management*** | ***Goals***  Build acceptance about HIV.  Prevention Information, motivation and behavior skills(Zhang et al., 2021)  To be confident about to disclose HIV in front of family.  HIV status disclosure with partners (Klein et al., 2013).  Reduce risky behavior (Tobin et al., 2017). |
| 8 | ***Relapse Prevention*** | ***Goals***  Implement relapse prevention strategies for managing possible future problems (Newcomb et al., 2015).  Discuss signs of lapse and relapse and strategies for continuing progress(Brandt et al., 2018).  Helping clients to develop coping plans in order to deal with these situations(Newcomb et al., 2015).  Analyze recovery in client(Newcomb et al., 2015). |
